# Leadership in public health crisis: a review to summarize lessons learned from COVID-19 pandemic: Angelica Valz Gris

**DOI:** 10.1093/eurpub/ckac129.544

**Published:** 2022-10-25

**Authors:** A Valz Gris, MR Gualano, T Osti, L Villani, VF Corona, F D'ambrosio, M Lomazzi, Collaborating Group Leadership Coalition, F Cascini, F Favaretti, W Ricciardi

**Affiliations:** 1 Department of Life Sciences and Public Health, Università Cattolica del Sacro Cuore, Rome, Italy; 2 Department of Public Health Sciences, University of Turin, Turin, Italy; 3 World Federation of Public Health Association, Geneve, Switzerland; 4 Center for Leadership in Medicine Research, Università Cattolica, Rome, Italy

## Abstract

**Background:**

During the COVID-19 pandemic, several public health challenges were faced, requiring worldwide leaders able to direct, guide, and establish appropriate strategies. The aim of this review was to summarize evidence on public health leadership during the COVID-19 era.

**Methods:**

The systematic literature review was conducted according to the PRISMA 2020 checklist. A search of relevant articles was performed in the PubMed, Scopus, and Web of Science databases. Eligible articles were any type of publication, published between 2020 and 2022, that outlined one or more characteristics of effective public health leadership during the COVID-19 pandemic We excluded all articles that did not explicitly address the COVID-19 pandemic or had a different setting.

**Results:**

A total of 2499 records were screened, and 45 articles were included. We identified 93 characteristics, clustered in six groups, that were reported as fundamental to be an effective leader in public health crises worldwide. Emotional intelligence and human traits (reported by 46.67% of the articles) were considered essential to build trust in the population and ensure cooperation with working groups. Communication skills (47%) are considered necessary to enable people to understand and accept measures. A supportive, multidisciplinary team and accountability mechanisms (33,33%) were highlighted as central elements, especially in the international field, to ensure reliability and consistency in action. Management skills (35,56%), adaptability (44,44%), and evidence-based approach (33,33%) were reported as key capabilities to ensure a prompt and rapid response to the challenges created by the pandemic.

**Conclusions:**

The identification of the attributes of an effective public health leader conducted in this study is useful in choosing the key personalities who must lead public health today and in the training of tomorrow's European and worldwide leaders to be ready to face future threats.

**Key messages:**

• Effective public health leaders in crisis are empathetic and trustworthy people, who have developed management and communication skills, and are able to make timely and evidence-based decisions.

• In order to create leaders capable of facing future threats, more emphasis in the training of public health workforce on soft skills and management competencies should be recommended.

